# Detection of human parvovirus 4 viremia in the follow-up blood samples from seropositive individuals suggests the existence of persistent viral replication or reactivation of latent viral infection

**DOI:** 10.1186/s12985-015-0326-0

**Published:** 2015-06-19

**Authors:** Mao-Yuan Chen, Chien-Ching Hung, Kuang-Lun Lee

**Affiliations:** Department of Internal Medicine, National Taiwan University Hospital, No. 7, Chung Shan South Road, 1000 Taipei, Taiwan

**Keywords:** Parvovirus 4, Persistent PARV4 infection, Latent PARV4 infection, Anti-PARV4 IgM

## Abstract

**Background:**

The transmission routes for human parvovirus 4 (PARV4) infections in areas with high seroprevalence are not known. In the work described here, persistent PARV4 viral replication was investigated by conducting a longitudinal study.

**Methods:**

Ten healthcare workers each provided a blood sample at the beginning of the study (first sample) and 12 months later (second sample). The paired samples were tested for PARV4-positivity by immunoblotting analysis and nested polymerase chain reactions.

**Results:**

IgG antibodies against PARV4 were detected in six participants, three of whom also had IgM antibodies against PARV4. The immunoblotting results did not vary over time. PARV4 DNA was detected in the first blood sample from one participant who had IgG antibodies against PARV4 and in the second blood samples from 2 participants who had IgG and IgM antibodies against PARV4.

**Conclusions:**

Detection of PARV4 DNA in the second blood samples from two seropositive participants suggests the existence of persistent PARV4 replication or reactivation of inactive virus in the tissues. The finding of persistent or intermittent PARV4 replication in individuals with past infections provides an important clue toward unraveling the non-parenteral transmission routes of PARV4 infection in areas where the virus is endemic.

## Background

In the subfamily *Parvovirinae* of the family *Parvoviridae*, members of four genera are currently known to infect humans, namely parvovirus B19 (B19V, genus *Erythroparvovirus*), human bocavirus (genus *Bocaparvovirus*), adeno-associated viruses (genus *Dependoparvovirus*) and human parvovirus 4 (PARV4, genus *Tetraparvovirus*); of these, the former two viruses can cause human diseases [1]. PARV4 was discovered in an intravenous drug user with an acute viral infection syndrome [2]. However to date, the clinical manifestations of PARV4 infection remain largely unknown. The seroprevalence of PARV4 varies geographically, but antibodies against PARV4 have been detected in 24.8 % of the general population in Cameroon in contrast to none of the general population in the United Kingdom [3]. Early serological studies supported a predominantly parenteral route for PARV4 infection in northern Europe because the seropositivity rates were 0 % and 0.9 % in the study groups where a risk of parenteral infection was not found [4, 5]. By contrast, recent studies found that 9.4 % of a low-risk group in Lithuania and 4.76 % of United Kingdom blood donors tested positive for PARV4 antibodies [6, 7]. How the study subjects in these two reports [6, 7] acquired PARV4 infection is not known.

The strong immune response induced by acute B19V infection can eliminate B19V viremia. Persistent B19V viremia does exist [8] but is infrequently found and characterized by a low viral load. However, B19V DNA frequently remains detectable in tissues long after viral exposure, perhaps even after a lifetime [9]. Similarly, PARV4 viremia was estimated to last only between 32 and 104 days in study subjects who displayed seroconversion [10] and PARV4 DNA could also be detected in 15 to 41 % of the liver, myocardium, lung and kidney tissues from non HIV-infected individuals in the absence of viremia which suggests persistence of viral DNA in tissues [11, 12]. Not every study found PARV4 DNA in tissue samples from non HIV-infected study subjects [13], although B19V and PARV4 DNA were detected in 54 % and 71 % of autopsy tissue samples, respectively, from HIV-infected study subjects in the same study [13]. However, both B19V and PARV4 DNA were absent in the plasma of 36 previously untreated HIV-infected patients despite the high probability of viral DNA persistence in tissue samples [13]. These findings raise the question of whether these viruses in tissues have the ability to actively replicate. In addition, the detection rate of B19V DNA in plasma was low (0.9 %) in blood donors from the United Kingdom despite 60.5 % of them being positive for anti-B19V IgG [14]. This suggests that most of the blood donors with past B19V infections do not have active B19V replication in their tissues or have replication levels below detection limit. In the case of PARV4, it was reported that the PARV4 DNA detection rate in blood donors from Los Angeles (USA) was low (2 %); however, the PARV4 seropositive rate is not known [15]. Although no direct evidence to date has supported persistent PARV4 replication in the host, the observation of sustained T cell responses against PARV4 without viremia suggests PARV4 may persist at levels below detection limit [10, 16].

In our previous report, the seven sequential blood samples collected from one HIV-infected male over a period of 21 months all tested positive for anti-PARV4 IgM but PARV4 DNA was found only in the second blood sample [17]. The patient might have had persistent PARV4 viremia, but the viral load was below the detection limit of the test method as has been reported previously in cases of persistent B19V viremia [8]. However, the possibility of re-infection could not be excluded because this was an immune-compromised host. To investigate the existence of PARV4 replication in healthy individuals with past PARV4 infections, we conducted the present study.

## Methods

### Study group

In this longitudinal study, ten healthcare workers (nine females) in our AIDS Center (National Taiwan University Hospital, Taiwan) donated blood on two occasions, with each donation 12 months apart. Their ages at the time of the first donation are listed in the Table. For each participant, 5 ml of blood was collected in an EDTA tube and the plasma was stored at −30 °C. The PARV4 serological status of these participants was unknown to us when they enrolled in the study. The investigation was carried out in accordance with the principles of the Declaration of Helsinki, and all of the participants provided informed consent. The collections of blood samples were approved by the ethics committee of the National Taiwan University Hospital.

### Immunoblotting and nested polymerase chain reaction (PCR)

Antibodies against PARV4 were detected using an immunoblotting method developed by our group [17]. Two of the recombinant fusion proteins used, SUMOVP2 (amino acids 272–630 of the translated PARV4 open reading frame, ORF2) and SUMOVP3 (amino acids 604–914 of the translated ORF2), and the control protein have been described in our previous study [17]. Here, an additional fusion protein SUMOVP1 (amino acids 1–275 of the translated ORF2) was generated by the methods used in our previous report [17]. The immunoblotting assays were analyzed by two independent researchers (blinded), and samples with reactivity to fusion protein VP1, VP2, or VP3 were considered seropositive.

PARV4 DNA was detected using nested PCR. Viral DNA was extracted from 200 μL of plasma, using the High Pure Viral Nucleic Acid Kit (Roche, Mannheim Germany). The elution volume was 50 μL, and 5 μL was used in each reaction. The PARV4 primers (designed to detect PARV4 genotype 2) have the following sequences: forward: 5′-ATTTCTGTAGCTCCACCAGGAGC-3′ (nucleotide, nt.316 to 338, the numbers represent the nucleotide position in ORF2 and ATG is nt. 1), reverse: 5′-CCATAAACCTCTAATAGCATTGCC-3′ (nt.903 to 880), inner forward: 5′- CAAAAGCACCAAATGCTGAAAG-3′ (nt.380 to 401), and inner reverse: 5′- GGGAATCAGCTCCTTCATCGCG-3′ (nt.610 to 589). The thermo-cycling conditions for nested PARV4 PCR in the first and second rounds were 94 °C for 2 min, followed by 36 cycles of 94 °C for 30 s, 54 °C for 30 s, and 72 °C for 30 s with a final extension of 7 min at 72 °C. PCR products were validated by sequencing and each DNA sample was tested using nested PCR six times, with the PARV4 ORF1 DNA template being included in each of 12 PCR experiments in this study as a negative control. The PCR experiments were repeated because it has been shown that PCR replicates are required to identify persistent B19V viremia when the B19V viral load is lower than the detection limit [8]. When six PCR replicates were performed on DNA samples with concentrations of 1, 0.5 or 0.25 PARV4 plasmid copies/μL, the number of positive results amounted to 6, 2 or 0, respectively.

## Results and discussion

**Fig. 1:**
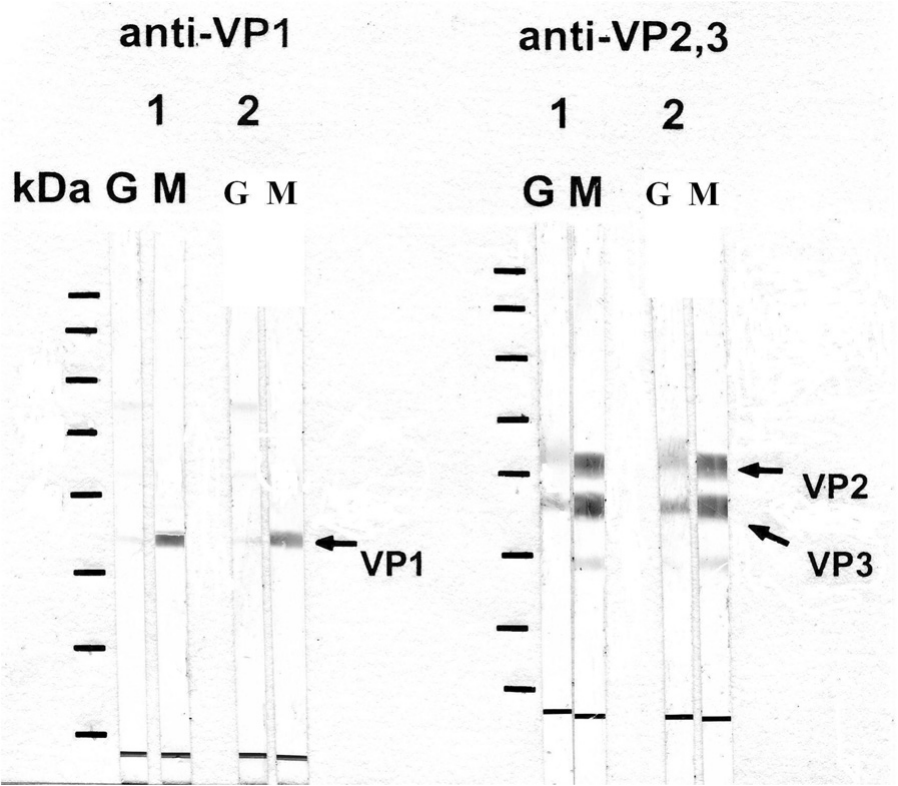
PARV4 immunoblots from study participant No. 10. The two blood samples collected from this subject 12 months apart are represented by 1 and 2. PARV4 DNA was detected in the second but not the first blood sample. Note the strong IgM and weak IgG reactivity over the study period. VP1 represents the fusion protein SUMOVP1 (a.a. 1–275 of the translated PARV4 open reading frame, ORF2); VP2 represents SUMOVP2 (a.a. 272–630 of translated ORF2) and VP3 represents SUMOVP3 (a.a. 604–914 of translated ORF2). The molecular weight markers (kDa) are 170, 130,100, 70, 55, 40, 35 and 25 from top to bottom of each blot. G: IgG; M: IgM; a.a.: amino acid

**Fig. 2:**
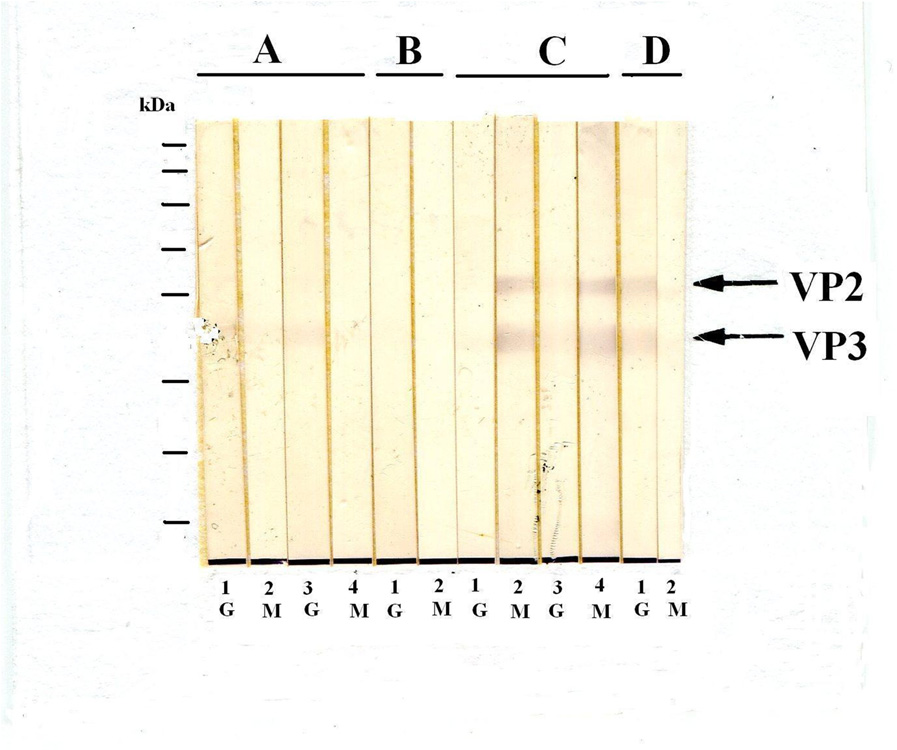
Immunoblots of participants No. 7 (**a**), 1 (**b**), 9 (**c**) and 5 (**d**). Immunoblots of the first blood samples are l (IgG) and 2 (IgM), immunoblots of the second blood samples are 3 (IgG) and 4 (IgM). For participants 1 and 5, the immunoblots of the second blood samples are not shown. Participant 1 was negative for anti-PARV4 IgG and IgM, participants 7 and 5 were positive for anti-PARV4 IgG and participant 9 was positive for anti-PARV4 IgG and IgM. Note that the anti-PARV4 IgG reactivities of participant 7 and 9 are weak and can hardly be detected after scanning. Anti-PARV4 VP1 is not shown. VP2, VP3 and molecular weight markers are the same as in Fig. 1

The serological and PCR results from the longitudinal study are listed in Table 1. Notably, the immunoblotting results for the 10 study participants in this study did not change over time. Anti-PARV4 IgM persisted without weakening in all three of the IgM-positive participants and IgG reactivity also remained constant (participant 9 and 10 are shown in Figs. 1 and 2, respectively). The IgG reactivity of participant 9 was so weak that it could hardly be detected after scanning (Fig. 2). One blood sample collected from participant 9 at 32 months before the start of the study also had the same immunoblotting results (strip not shown). We cannot explain the persistence of IgM reactivity with weak IgG reactivity noted in participants 9 and 10 (both were PARV4 DNA-positive) but this pattern is not uncommon in our experience and these subjects were likely to be diagnosed as having current PARV4 infection without knowledge of their preceding immunoblotting results. Interestingly, the PARV4 DNA-positive participant (number 7) who was negative for anti-PARV4 IgM also had weak IgG reactivity (Fig. 2). PARV4 DNA was detected only in the first sample (one participant) or in the second sample (two participants). The number of positive PCR replicates was one (two samples) or 2 (one sample). The three nucleotide sequences of the PCR products are identical to the corresponding region of the GenBank sequence GU120197.1.

**Table 1:** Longitudinal serological and PCR data

Participant	Sex	Age	Sample	Anti-PARV4 immunoglobulin	No. of positive results in six PCR replicates
1	M	38	1	Negative	0/6
			2	Negative	0/6
2	F	45	1	Negative	0/6
			2	Negative	0/6
3	F	24	1	Negative	0/6
			2	Negative	0/6
4	F	36	1	Negative	0/6
			2	Negative	0/6
5	F	26	1	IgG positive	0/6
			2	IgG positive	0/6
6	F	32	1	IgG positive	0/6
			2	IgG positive	0/6
7	F	28	1	IgG weakly positive	1/6
			2	IgG weakly positive	0/6
8	F	25	1	IgG & IgM positive	0/6
			2	IgG & IgM positive	0/6
9	F	30	1	IgG weakly positive & IgM positive	0/6
			2	IgG weakly positive & IgM positive	2/6
10	F	24	1	IgG & IgM positive	0/6
			2	IgG & IgM positive	1/6

*M* male, *F* female, *No*. number

Both B19V and PARV4 viruses have two capsid proteins, VP1 and VP2 [18], and antibodies against linear B19V VP2 epitopes have been shown to disappear 6 months after acute B19V infection [19]. In the present study, the three PARV4-SUMO fusion proteins SUMOVP1, SUMOVP2 and SUMOVP3 represent the PARV4 VP1-unique sequence, the PARV4 VP1-VP2 overlapping region, and the carboxyl terminal region of PARV4 VP2, respectively. In contrast to B19V, the antibodies against the linear epitopes of PARV4 VP2 did not disappear over time (Figs. 1 and 2).

To date, studies on the anti-PARV4 IgM response have yielded inconsistent results. A monthly follow-up study of intravenous drug users found that five drug users had seroconversion to PARV4, but four of them were positive for anti-PARV4 IgM only in the blood sample collected at seroconversion; the anti-PARV4 IgM became undetectable in the blood sample collected after a month [10]. For the one exception, two sequential blood samples tested positive for anti-PARV4 IgM, and the duration of the IgM response was 32–93 days. All the IgM-positive blood samples collected at seroconversion had detectable PARV4 DNA, but viremia disappeared in the second IgM-positive blood sample collected after one month and the duration of viremia was estimated to have lasted between 32 and 98 days. This result suggested that anti-PARV4 IgM and PARV4 viremia were both short-lived and the existence of PARV4 DNA was expected in most of the IgM-positive blood samples. In contrast, a serological study conducted in Finland found that four (5.1 %) HIV-infected intravenous drug users tested positive for anti-PARV4 IgM without having PARV4 viremia [5]. Furthermore, the presence of high-avidity anti-PARV4 IgG indicated that the four drug users had previous immunity rather than primary infection. Additionally, the anti-PARV4 IgM-positive rate was 7 % in a Lithuanian study on patients with acute respiratory disease in contrast to 0.9 % in a Finnish study on university students [5, 6]. These IgM-positive Lithuanian patients were grouped into current (IgM+, IgG–) ongoing, or recent PARV4 infection groups (IgM+, IgG+); however, PARV4 DNA was not detectable in any of the IgM-positive blood samples. In our experience, IgM antibodies against PARV4 can remain detectable for 12 months or longer, and the high IgM-positive rates are explainable by a prolonged IgM response, but PARV4 DNA might not have been detected in IgM-positive blood samples without conducting PCR replicates.

In the present study, the possibility of low-level viral infections in some subjects is suggested by the infrequent positive results of the PCR replicates, but false positive results seem unlikely because not a single negative control was positive (a total of 12 negative control reactions were performed in this study). Furthermore, the PARV4 DNA-positive blood samples in our PARV4 studies to date have always shown PARV4 antibody positivity with only one exception; this uneven distribution conflicts with the false positive results. A low viral load in the presence of anti-PARV4 IgG without IgM was noted in one participant (number 7 in the table), this was considered to result from persistent viral replication in a B19V study of blood donors [14]. Our study does not demonstrate persistent viral replication directly because PARV4 viremia was not found at more than one time point. Nevertheless, the detection of PARV4 DNA in the second of the two blood samples from two participants who were positive for anti-PARV4 IgM and IgG strongly suggests the existence of persistent PARV4 replication for the following reasons. (1) Primary PARV4 infection can be excluded because of the pre-existing anti-PARV4 IgM and IgG, which suggests the possibility of persistent PARV4 replication, reactivation of latent PARV4 infection or re-infection. (2) Re-infection can boost the pre-existing IgG antibody response, but this was not observed in the two study participants. (3) The low (1/35) seropositive rate in siblings of PARV4-infected hemophiliac patients [4] and the low PARV4 seroprevalence in United Kingdom blood donors [7] suggest that PARV4 is not easily transmitted by person-to-person contact through non-parenteral routes hence the chance of re-infection is low. (4) It is possible that persistent low-level viral replication below the detection limit existed in the first blood samples, but reappearance of viremia due to reactivation of inactive virus in the tissues is also possible.

## Conclusions

The three PARV4 DNA-positive participants in this study were females of child-bearing age and persistent PARV4 replication is a possibility for them. If they become pregnant, PARV4 virus may be transmitted to their newborns, as was shown in our previous study [20]. Trans-placental PARV4 infection may be one of the major transmission routes in Taiwan because PARV4 seroprevalence in healthcare workers is high (43.6 %) [17], and 26 % of the seropositive individuals in our study of 31 healthcare workers were shown to have detectable PARV4 DNA by the method described herein (Chen MY, unpublished data). The hypothesis is also in agreement with the high PARV4 DNA-positive rates observed in Ghanaian infants and children [21]. To address this issue, prospective studies on PARV4 infection in pregnant women and their newborns in PARV4 endemic areas are required.
